# Comparison of different obesity indices associated with type 2 diabetes mellitus among different sex and age groups in Nantong, China: a cross-section study

**DOI:** 10.1186/s12877-021-02713-w

**Published:** 2022-01-03

**Authors:** Qiwei Ge, Min Li, Zhengcheng Xu, Zhigang Qi, Huiyan Zheng, Yuxin Cao, Hao Huang, Xiaoyang Duan, Xun Zhuang

**Affiliations:** 1 Department of Epidemiology and Medical Statistics, School of Public Health, Nantong University, No 9 Seyuan Road, Nantong, Jiangsu 226019 China; 2 Nantong Chongchuan District Center for Disease Control and Prevention, Nantong, Jiangsu 226000 China

**Keywords:** Obesity, Anthropometric indices, Type 2 diabetes mellitus, Chinese

## Abstract

**Background:**

Obesity is associated with type 2 diabetes mellitus (T2DM). However, the obesity index that is most closely related to type 2 diabetes remains controversial. Therefore, the aim of this study was to compare the associations of five anthropometric indices (body mass index [BMI], body adiposity index, waist circumference [WC], waist-to-hip ratio, and waist-to-height ratio [WHtR]) with T2DM among Chinese adults divided into four groups according to sex and age.

**Methods:**

A total of 4007 adult participants (1669 men and 2338 women) were included in the study. Odds ratios (ORs) and 95% confidence intervals were used with binary logistic regression models to estimate the risk of T2DM for each obesity index. Furthermore, we compared the area under the receiver operating characteristic curve (AUC) of each obesity index for the criterion of T2DM under the influence of risk factors.

**Results:**

WC had the highest OR (3.211 and 1.452) and AUC (0.783 and 0.614) in both age groups of men. However, WHtR (OR = 2.366, AUC = 0.771) and BMI (OR = 1.596, AUC = 0.647) were the optimal criteria for predicting T2DM among females in the 18–59 and ≥ 60 years age groups, respectively.

**Conclusions:**

This study suggests that there is a positive association between obesity-related anthropometric indices and T2DM in different sex and age groups. WC appears to be the optimal anthropometric index for predicting T2DM in men. The optimal obesity indices related to T2DM were WHtR and BMI for women aged 18–59 and ≥ 60 years, respectively.

## Background

Type 2 diabetes mellitus (T2DM), the most common form of diabetes, accounts for more than 96% of all diabetes cases [1]. In China, the prevalence of T2DM has increased to 11.6% [2], affecting more than 100 million adults [3]. Previous studies have shown that weight gain is an independent risk factor for diabetes, while abdominal obesity is associated with T2DM [4–6]. Anthropometric indices used to define obesity can help in the identification of individuals or groups of people with a variety of specific health thresholds [7]. Commonly used anthropometric indices of obesity are body mass index (BMI), body adiposity index (BAI), waist circumference (WC), waist-to-hip ratio (WHR), and waist-to-height ratio (WHtR) [8].

Some studies have been conducted to assess the relationship between anthropometric obesity indices and T2DM [9, 10]; however, the results of these studies are inconsistent. A cross-sectional study in the population of northern Iran showed that while WHR (area under the curve [AUC] = 0.7303 in men, AUC = 0.7529 in women) had an appropriate discriminatory capability for T2DM, BAI and BMI did not [11]. Another hospital-based cross-sectional study conducted among 363 consecutively selected patients with T2DM showed that WHtR (AUC = 0.85) and WC (AUC = 0.79) were the optimal predictors of metabolic syndrome in women and men, respectively [12]. Similar studies have been conducted in China, but unfortunately, the results are inconsistent. A study of people aged > 50 years in Jinan showed that the optimal indicator of the relationship between obesity and T2DM is WHtR (OR = 2.572) and BMI (OR = 2.764) for men and women, respectively [13]. However, another study in Changchun showed that WHtR (AUC = 0.628 in men, AUC = 0.676 in women) is the optimal index for predicting T2DM in Chinese adults [14].

The inconsistency of the optimal indicators obtained in previous studies may be related to ethnic and regional differences [15–18]. In addition, previous studies have stratified studies by age or sex without cross-grouping, and the association between obesity indicators and T2DM in different sex and age groups remains unclear. Therefore, this study aimed to compare the associations of five anthropometric indices (BMI, BAI, WC, WHR, and WHtR) with T2DM among Chinese adults divided into four groups according to sex and age and to supplement similar studies in different regions of China. The thresholds of these anthropometric indices for obesity were also evaluated as reasonable predictors of T2DM.

## Methods

### Study design and participants

The participants in this study were adults who were enrolled from the baseline survey of the Chronic Disease Study in 2018, which was a cross-sectional study based on urban community-dwelling residents in the Chongchuan district with an estimated 720,000 permanent residents in Nantong City, Jiangsu Province, China.

**Fig. 1:**
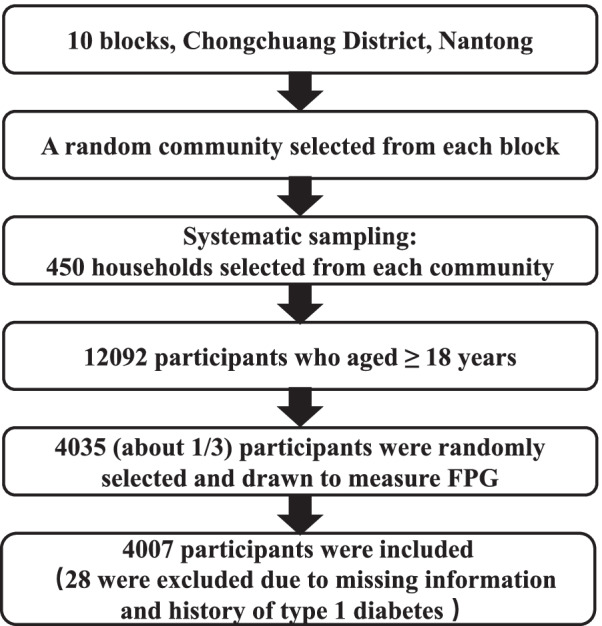
The sample diagram of the study object

In the first stage of enrollment, all 10 blocks of the Chongchuan district were selected and one residential community was randomly selected from each of the blocks. In the second stage, systematic sampling was conducted to select 450 households in each residential community. In the third stage, residents who aged ≥18 years and had lived in the district for at least 1 year were selected from each household. Finally, 12,092 people from 4500 households were recruited to complete the questionnaires and undergo the relevant physical examinations. In total, 4035 participants (about 1/3) were randomly selected and blood was drawn to measure fasting plasma blood glucose (FPG). Twenty-eight people were excluded because they provided no information about weight, height, WC, or hip circumference or had a history of type I diabetes, leaving a total of 4007 participants for the final analyses (Fig. 1).

### Sample size evaluation

We calculated the minimum sample size required by using the following formula [19]:

$$n={z}_{1-a/2}^2\times p\left(1-p\right)/{d}^2$$

In this formula, ${z}_{1-a/2}^2$ =1.96 ≈ 2 at 5% type I error; *p*, which is the prevalence of T2DM among Chinese adults, was approximately 10% [20], and d, which is the absolute error or precision, was 10% *p* in our study. In addition, a multi-stage random sampling method was performed in this study. Therefore, the minimum sample size was 3600. The final sample size selected for analysis was sufficiently large compared with the number we calculated.

$$n=400\times \frac{q}{p}=400\times \frac{0.9}{0.1}=3600$$

### Data collection

A trained investigator from the Center for Disease Control and Prevention of the Chongchuan district in Nantong and the School of Public Health in Nantong University interviewed each participant using standardized questionnaires and collected information on demographic data (age, sex, education, etc.), lifestyle behaviors (such as smoking, alcohol consumption, etc.), and family disease history (hypertension, diabetes).

### Physical examination

Anthropometric indices were measured by trained staff who followed standard procedures. Height was measured using a standard stadiometer, and each participant underwent measurement without shoes. Weight was measured to the nearest 0.1 kg using an electronic scale, with the subjects wearing light indoor clothing. WC was measured to the nearest 0.1 cm at the midpoint between the lowest rib margin and the level of the anterior superior iliac crest using a flexible anthropometric tape. Hip circumference (HC) was measured to the nearest 0.1 cm at the greatest protrusion of the gluteal muscles. Participants were required to designate a hospital for measurement of FPG.

### Ethics approval

This study was conducted in accordance with the World Medical Association Declaration of Helsinki: ethical principles for medical research involving human subjects and was approved by the Ethics Committee of The Third People’s Hospital of Nantong City (No.: EK2018009; 18 June, 2018). Written informed consent was obtained from each participant after they were informed of the objectives and benefits of this study.

### Definitions of variables

Patients who had T2DM were defined as those who had been diagnosed in a secondary hospital or who had FPG of ≥7.0 mmol/L in the fasting blood glucose testing [21]. BMI was calculated as weight (kg) divided by height squared (m^2^). According to “the standards of the Chinese guidelines for the prevention and control of overweight and obesity in adults”, BMI of ≥24 and ≥ 28 kg/m^2^ were regarded as overweight and obese, respectively [22]. BAI was measured using the following formula [hip circumference (cm)/height^1.5^ (m)] – 18, BAI ≥ 25.5 and ≥ 29.0 for men, ≥30.5 and ≥ 35.0 for women was regarded as overweight and obese, respectively [23]. WC of ≥90 cm for men and ≥ 80 cm for women were defined as central obesity. WHR was calculated as WC (cm) divided by HC (cm), with values of > 0.9 for men and > 0.8 for women indicating central obesity. WHtR was calculated as WC (cm) divided by height (cm) and values of ≥0.5 indicated obesity [22].

### Statistical analysis

All quantitative variables are described as mean ± standard deviation (SD) for normal distributions, whereas qualitative data were described as percentages. Student’s t-test and chi-square test were performed to estimate the difference between the quantitative and qualitative data of different groups, respectively. The following weight variables were estimated in this study: BMI, BAI, WC, WHR, and WHtR. Odds ratios (ORs) and 95% confidence intervals (CIs) were used with binary logistic regression models to estimate the risk of T2DM for each obesity index.

For each index, receiver operating characteristic (ROC) curve analysis was used to identify the value with the Youden index as a predictor of T2DM. Statistical significance was set at *P*-value of < 0.05. Statistical analyses were performed using SPSS 24.0 for Windows.

## Results

The demographic data of the 4007 eligible participants included in this study are shown in Table 1. The mean age of the participants was 46.78 ± 14.95 years, and at the age of 60, the population was divided into two groups. Naturally, the prevalence of T2DM increases with age. The ratio of male to female participants was 1: 0.71, and men had a higher risk of T2DM than women. Regarding education, participants who had a primary school or lower education accounted for the highest percentage (44.1%). The higher the education level, the lower the T2DM risk. In addition, smoking status, alcohol drinking status, positive family history of diabetes and hypertension were significantly correlated with T2DM. Finally, there was a statistically significant difference between participants who had T2DM and those who did not with respect to BMI, WC, and WHtR.

**Table 1:** Distribution of sociodemographic and lifestyle characteristics of participants

Variable	Total (*n* = 4007)	No diabetes (*n* = 3376)	Diabetes (*n* = 631)	OR (95%CI)	P
Age	46.78 ± 14.95	46.36 ± 15.34	54.4 ± 9.54		< 0.001
18-	3376(84.3)	1390(94.0)	1986(78.5)	1	
60-	631(15.7)	88(6.0)	543(21.5)	4.319(3.413–5.465)	< 0.001
Sex					
Male	1669(41.7)	1382(40.9)	287(45.5)	1	
Female	2338(58.3)	1994(59.1)	344(54.5)	0.831(0.700–0.986)	0.034
Education					
Primary school and lower	1236(30.8)	958(28.4)	278(44.1)	1	
Secondary school	1252(31.2)	1050(31.1)	202(32.0)	0.663(0.542–0.811)	< 0.001
High secondary school	838(20.9)	727(21.5)	111(17.6)	0.526(0.414–0.669)	< 0.001
College or university	386(9.6)	360(10.7)	26(4.1)	0.249(0.164–0.379)	< 0.001
Bachelor and higher	295(7.4)	281(8.3)	14(2.2)	0.172(0.099–0.298)	< 0.001
Smoking					
No	3479(86.8)	2938(87.0)	541(85.7)	1	
Yes	446(11.1)	380(11.3)	66(10.5)	0.943(0.715–1.244)	0.679
Quit	82(2.0)	58(1.7)	24(3.8)	2.247(1.384–3.648)	0.001
Alcohol drinking					
No	3224(80.5)	2723(80.7)	501(79.4)	1	
Yes	726(18.1)	612(18.1)	114(18.1)	1.012(0.811–1.263)	0.913
Quit	57(1.4)	41(1.2)	16(2.5)	2.121(1.181–3.809)	0.012
Sweet tooth					
No	3844(95.9)	3232(95.7)	612(97.0)	1	
Yes	163(4.1)	144(4.3)	19(3.0)	0.697(0.429–1.133)	0.145
Physical activity					
No	1969(49.1)	1658(49.1)	311(49.3)	1	
Yes	2038(50.9)	1718(50.9)	320(50.7)	0.993(0.838–1.177)	0.935
Family history					
No	3708(92.5)	3192(94.5)	516(81.8)	1	
Yes	299(7.5)	184(5.5)	115(18.2)	3.866(3.008–4.969)	< 0.001
Hypertension					
No	2721(67.9)	2360(69.9)	361(57.2)	1	
Yes	1286(32.1)	1016(30.1)	270(42.8)	1.737(1.422–2.054)	< 0.001
BMI, kg/m^2^					
Normal	2064(51.5)	1801(53.5)	258(41.0)	1	
Overweight/obesity	1941(48.5)	1570(46.5)	371(59.0)	1.650(1.388–1.961)	< 0.001
WC, cm					
Normal	1829(45.6)	1603(47.5)	226(35.8)	1	
Overweight/obesity	2178(54.4)	1773(52.5)	405(64.2)	1.620(1.358–1.932)	< 0.001
BAI					
Normal	2591(64.7)	2253(66.7)	338(53.6)	1	
Overweight/obesity	1416(35.3)	1123(33.3)	293(46.4)	1.739(1.464–2.066)	< 0.001
WHR					
Normal	972(24.3)	831(24.6)	141(22.3)	1	
Overweight/obesity	3035(75.7)	2545(75.4)	490(77.7)	1.135(0.926–1.390)	0.222
WHtR					
Normal	2528(63.1)	2206(65.3)	322(51.0)	1	
Overweight/obesity	1479(36.9)	1170(34.7)	309(49.0)	1.809(1.524–2.148)	< 0.001

*P* < 0.05, statistically significant

Considering the confounding effect of age and sex, we divided the participants into four subgroups based on both variables (Table 2). In all four subgroups, people with T2DM had higher WC than the controls (*P* < 0.05) and WHR was not significantly different between the T2DM group and the controls (*P* > 0.05). However, in men, BMI was not significantly different between the T2DM group and the controls (P > 0.05), whereas in the female group, BMI was comparatively higher in the T2DM group. Moreover, BAI and WHtR were higher in the T2DM group, excluding men aged ≥60 years.

**Table 2:** Anthropometric measurement characteristics of participants

Variable	No T2DM	T2DM	*P*	Variable	No T2DM	T2DM	*P*
Male (age:18–59)				Female (age:18–59)			
BMI (kg/m2)	24.11 ± 2.72	25.78 ± 2.92	0.053	BMI (kg/m2)	23.21 ± 2.90	25.06 ± 3.19	< 0.001
BAI	25.01 ± 4.50	27.26 ± 4.86	0.048	BAI	26.62 ± 4.27	29.28 ± 3.60	< 0.001
WC (cm)	85.71 ± 7.90	88.74 ± 7.84	0.021	WC (cm)	80.14 ± 6.63	84.53 ± 7.87	< 0.001
WHR	0.89 ± 0.06	0.88 ± 0.06	0.591	WHR	0.87 ± 0.06	0.87 ± 0.05	0.990
WHtR	0.49 ± 0.05	0.52 ± 0.04	0.001	WHtR	0.49 ± 0.04	0.52 ± 0.05	< 0.001
Male (age: ≥60)				Female (age: ≥60)			
BMI (kg/m2)	24.27 ± 3.39	24.66 ± 3.32	0.112	BMI (kg/m2)	24.12 ± 3.14	25.18 ± 4.16	< 0.001
BAI	25.45 ± 5.43	25.61 ± 4.83	0.536	BAI	29.08 ± 4.83	30.09 ± 4.86	0.001
WC (cm)	86.54 ± 7.83	87.84 ± 8.52	0.033	WC (cm)	83.46 ± 8.08	85.48 ± 7.84	< 0.001
WHR	0.90 ± 0.05	0.91 ± 0.13	0.147	WHR	0.88 ± 0.06	0.89 ± 0.10	0.334
WHtR	0.51 ± 0.05	0.51 ± 0.05	0.052	WHtR	0.52 ± 0.05	0.54 ± 0.05	< 0.001

*BMI* body mass index; *BAI* body adiposity index; *WC* waist circumference; *WHR* waist-to-hip ratio; *WHtR* waist-to-height ratio; *T2DM* type 2 diabetes mellitus

Values are presented as mean ± SD

*P* < 0.05, statistically significant

The associations between the prevalence of T2DM and the obesity indices are shown in Table 3. In both groups of women, the findings indicated positive associations between the five obesity indices but WHR with T2DM, even after adjustments for risk-related factors in Model 2. The difference was that WHtR (2.366) had the highest OR value in the 18–59 age group, while BMI (1.596) was the highest in the ≥60 age group after adjusting for risk factors. However, the associations in the male groups compared with the female groups were completely different. In men aged 18–59 years, BAI, WC and WHtR were positively associated with T2DM, and the highest value of OR (3.211) was observed for WC in Model 2. In men aged ≥60 years, only WC had a positive association with T2DM, and the OR was 1.478 and 1.452 for Model 1 and Model 2, respectively.

**Table 3:** Crude and adjusted ORs for T2DM in relation to anthropometric indices

Variable	Model 1 OR (95%CI)	Model 2 OR (95%CI)	Variable	Model 1 OR (95%CI)	Model 2 OR (95%CI)
Male (age:18–59)			Female (age:18–59)		
BMI (≥24 kg/m2)	1.492(0.780–2.855)	1.540(0.782–3.032)	BMI (≥24 kg/m2)	3.176(1.733–5.819) **	2.183(1.158–4.113) *
BAI (≥25.5)	2.184(1.146–4.161) *	2.178(1.112–4.267) *	BAI (≥30.5)	2.162(1.127–4.148) *	1.508(1.059–2.998) *
WC (≥90 cm)	3.699(1.912–7.156) **	3.211(1.625–6.346) **	WC (≥80 cm)	2.849(1.360–5.969) *	2.287(1.071–4.883) *
WHR (> 0.9)	0.900(0.475–1.704)	0.890(0.455–1.740)	WHR (> 0.8)	1.074(0.415–2.783)	0.814(0.306–2.168)
WHtR (≥0.5)	3.189(1.676–6.068) **	2.827(1.446–5.527) *	WHtR (≥0.5)	3.480(1.919–6.312) **	2.366(1.265–4.427) *
Male (age: ≥60)			Female (age: ≥60)		
BMI (≥24 kg/m2)	1.083(0.813–1.442)	1.110(0.827–1.490)	BMI (≥24 kg/m2)	1.746(1.290–2.172) **	1.596(1.200–2.052) **
BAI (≥25.5)	1.103(0.830–1.467)	1.070(0.796–1.438)	BAI (≥30.5)	1.395(1.177–1.806) *	1.278(1.078–1.671) *
WC (≥90 cm)	1.478(1.104–1.980) *	1.452(1.070–1.970) *	WC (≥80 cm)	1.703(1.226–2.366) **	1.575(1.124–2.208) **
WHR (> 0.9)	1.147(0.859–1.530)	1.104(0.822–1.484)	WHR (> 0.8)	1.637(0.855–3.134)	1.592(0.819–3.093)
WHtR (≥0.5)	1.225(0.916–1.637)	1.172(0.865–1.588)	WHtR (≥0.5)	1.323(1.025–1.709) **	1.203(1.023–1.568) *

*BMI* body mass index; *BAI* body adiposity index; *WC* waist circumference; *WHR* waist-to-hip ratio; *WHtR* waist-to-height ratio; *T2DM* type 2 diabetes mellitus; *OR* odds ratio

Model 1: unadjusted

Model 2: adjusted for education, lifestyle (physical activity, smoking, alcohol drinking, and sweet tooth), medical history characteristics (family history or hypertension), and time of diagnosis of type 2 diabetes mellitus

*: P < 0.05, **: *P* < 0.001, statistically significant

Furthermore, we compared the AUC of each obesity index for the prediction of T2DM with the adjustment of other risk factors such as education, smoking, alcohol consumption, exercise, sweet tooth, hypertension and family disease history. Moreover, we also considered that the patients may control their weight after the diagnosis of T2DM. As Table 4 shows, similar to the previous results, the AUC of BAI and WC were the largest (0.783 and 0.614) among the five anthropometric indices in the two groups of men. In addition, WHtR (AUC = 0.771) and BMI (AUC = 0.647) tended to be the best predictors of T2DM among females in the age group of 18–59 and ≥ 60 years, respectively.

**Table 4:** AUCs for anthropometric indices in relation to T2DM

Variable	AUC(95%CI)	Sensitivity	Specificity	Youden index
Male (age:18–59)				
BMI (kg/m^2^) + other factors^a^	0.748(0.677–0.819)	0.683	0.690	0.373
BAI + other factors	0.750(0.678–0.822)	0.854	0.545	0.399
WC (cm) + other factors	0.783(0.717–0.849)	0.829	0.627	0.456
WHR + other factors	0.730(0.657–0.803)	0.732	0.635	0.367
WHtR + other factors	0.768(0.701–0.836)	0.707	0.667	0.374
Male (age: ≥60)				
BMI (kg/m^2^) + other factors	0.586(0.543–0.628)	0.472	0.677	0.149
BAI + other factors	0.585(0.542–0.629)	0.439	0.719	0.158
WC (cm) + other factors	0.614(0.572–0.657)	0.593	0.611	0.204
WHR + other factors	0.585(0.542–0.628)	0.488	0.664	0.152
WHtR + other factors	0.593(0.550–0.636)	0.455	0.726	0.181
Female (age:18–59)				
BMI (kg/m^2^) + other factors	0.769(0.709–0.829)	0.936	0.473	0.409
BAI + other factors	0.744(0.674–0.813)	0.489	0.842	0.331
WC (cm) + other factors	0.759(0.696–0.821)	0.872	0.517	0.389
WHR + other factors	0.739(0.667–0.811)	0.468	0.872	0.340
WHtR + other factors	0.771(0.712–0.831)	0.660	0.753	0.413
Female (age: ≥60)				
BMI (kg/m^2^) + other factors	0.647(0.612–0.682)	0.532	0.685	0.217
BAI + other factors	0.624(0.587–0.660)	0.451	0.730	0.181
WC (cm) + other factors	0.634(0.599–0.669)	0.623	0.560	0.183
WHR + other factors	0.619(0.583–0.656)	0.687	0.482	0.169
WHtR + other factors	0.629(0.592–0.665)	0.515	0.682	0.197

*AUC* area under the curve; *BMI* body mass index; *BAI* body adiposity index; *WC* waist circumference; *WHR* waist-to-hip ratio; *WHtR* waist-to-height ratio; *T2DM* type 2 diabetes mellitus

^a^Other factors: education, lifestyle (physical activity, smoking, alcohol drinking, sweet tooth), medical history characteristics (family history or hypertension), and time of diagnosis of type 2 diabetes mellitus

## Discussion

In this study, we compared the associations of five anthropometric indices with T2DM among Chinese adults divided into four groups according to sex and age. Using survey data from a cross-sectional study of chronic diseases in Nantong City, East China, we found strong evidence of a positive correlation between obesity-related measures and T2DM. WC appeared to be the optimal anthropometric index for predicting T2DM in men. The optimal obesity indices related to T2DM were WHtR and BMI in women aged 18–59 and ≥ 60 years, respectively. This ascertains men and young women (18–59 years old) with T2DM should pay more attention to central obesity, while older women (≥60 years old) with T2DM should be more concerned with systemic obesity. In the future, this study should be integrated with relevant studies in other parts of China to obtain obesity indices suitable for predicting T2DM in China of different sexes and ages. Our results provide the most closely related obesity indices for T2DM in different sex and age groups, which will help other researchers to accurately evaluate the relationship between weight and T2DM.

The results of this study also show that on comparison with other obesity indicators, WHtR had a greater impact on the risk of T2DM in the general population, which is consistent with previous research results. Previous studies have shown that height has an important influence on diabetes. WC and WHR measurements do not consider the influence of height [24]. To overcome this defect, most researchers have used WHtR to study the relationship between overweight, obesity and chronic diseases [25, 26]. The WHtR was easy to calculate, and had a significant relationship with WC without differences for sex. Setting ≥0.5 as the cut-in point can predict well the risk of diabetes [27]. A study in China showed that WHtR did not affect on impaired fasting blood glucose, but it was related to diabetes. It can be used as an important screening index for the elderly [28].

In addition, central and systemic obesity indices showed different correlations in different sex and age groups. WC had the highest OR and the largest AUC before adjusting for confounding variables in both the age groups of men. The results of a meta-analysis of 16 cohort studies involving different ethnic groups in Asia showed that for every additional SD in BMI and WC, the risk of diabetes was 52 and 54%, respectively. WC is more closely related to diabetes [29]. A survey in China found that for every increase in SD of BMI and WC, the risk of diabetes increased by 53 and 64%, respectively. WC has a large impact on the risk of diabetes [30]. The conclusions of these studies were the same as those in the present study, which might be related to the fact that insulin resistance is an important factor for diabetes [31]. Studies have confirmed that insulin antagonism is more evident in central obesity, and WC is recognized as an important indicator for measuring central obesity, which can better reflect insulin resistance [32]. Therefore, it is recommended that the waist circumference can be reduced through appropriate physical exercise and other means based on weight control, thereby reducing the risk of T2DM.

However, among women, WHtR and BMI tended to be the optimal predictors of T2DM in the 18–59 and ≥ 60 age groups, respectively. The reason for the sex difference in the relationship between obesity indicators and T2DM may be related to sex differences in body fat distribution. Fat is mainly found as visceral fat in men and subcutaneous fat in women, which can provide evidence for the sex differences [33–35]. In thin Asian men with less subcutaneous fat, WC may be a better indicator of visceral obesity than BMI. Among thin Asian women, the impact of subcutaneous fat was greater than that of WC, and BMI may be more suitable as an indicator of overall fat and abdominal fat accumulation than WC [36–38]. In contrast, compared with women, men were more stressed at work and slept less. Working pressure can increase the epinephrine secretion level of the body, leading to more fat in the abdomen [39]. Sleep deprivation is closely related to cardiovascular risk factors such as inflammatory markers in the blood circulation; the resulting inflammatory response may be one of the mechanisms leading to metabolic diseases such as abdominal obesity and T2DM [40]. However, in recent years, Chinese women’s nutrition-rich diet, reduced exercise, and increasing pressure at work have led to an increase in central obesity in young and middle-aged women [41]. This may explain why obesity indices most closely associated with T2DM are inconsistent across age groups.

BAI was also included in this study because it is believed to be able to assess percent body fat more objectively based on hip circumference and height [42]. We found that BAI was positively associated with T2DM, except in men aged ≥60 years. The positive association of T2DM with BAI was not stronger than that observed in other commonly used obesity indices. Consistently, the capacity of BAI in predicting T2DM was also found to be lower than BMI, WC and WHtR, which are recognized as the most frequently used obesity indices. This was inconsistent with American research results stating that BAI was superior to BMI [43, 44], possibly because BAI was proposed by the research on hip circumference and height of African Americans and Mexican Americans [42], while the characteristics of height and hip circumference of Chinese Han people were quite different from them.

We also noticed no positive association between WHR and T2DM in all sex and age groups. Therefore, the use of WHR as a weight predictor of T2DM is not recommended. This finding is similar to that of a study conducted in Changchun. In that study, the cut-off value for WHR was not suitable for predicting T2DM compared with that of BMI and WHtR [19].

One of the strengths of the present study is that we randomly selected a community-based population with a broad age range, which increased the generalizability of our results to local populations. Furthermore, we compared the predictive values of five anthropometric indices (BMI, BAI, WC, WHR, and WHtR) to achieve an accurate evaluation of T2DM prediction by obesity indices for different age and sex groups. We believe that the findings of the present study may provide a reference for the selection of the appropriate anthropometric obesity indices for estimating the risk of T2DM in people of different sexes and ages.

The present study has several limitations. First, as a cross-sectional study, the causality between obesity indices and T2DM could not be accurately proven. Longitudinal follow-up studies are needed to verify the results of the present study. Second, participants who self-reported T2DM may alter their lifestyle habits such as diet and exercise to skew their anthropometric indices. Although this was considered and adjusted for in the model and ROC curve analyses, information migration was unavoidable. Finally, only residents from a single area were recruited for this study. Therefore, the conclusions of this study may have some limitations in extrapolation due to diet and lifestyle differences. Since we did not conduct external validation in other regions, it was difficult to extrapolate the results to all regions of China. In the future, further studies concerning other regions in China are needed to verify our results.

## Conclusions

This study depicted a positive association between obesity-related anthropometric indices and T2DM in different sex and age groups. WC appeared to be the optimal anthropometric index for predicting T2DM in men. The best obesity indices related with T2DM were WHtR and BMI, in women aged 18–59 and ≥ 60 years, respectively.

## Data Availability

The datasets generated during and analyzed during the current study are not publicly available, but are available from the corresponding author on reasonable request.

## Abbreviations

T2DM: type 2 diabetes mellitus
BMI: body mass index
BAI: body adiposity index
WC: waist circumference
WHR: waist-to-hip ratio
WHtR: waist-to-height ratio
AUC: area under the curve
ROC: receiver operating characteristic
FPG: fasting plasma blood glucose
IRAS: Insulin Resistance Atherosclerosis Study
SD: standard deviation

## References

[1] Saeedi P, Petersohn I, Salpea P, Malanda B, Karuranga S, Unwin N, et al. Global and regional diabetes prevalence estimates for 2019 and projections for 2030 and 2045: Results from the International Diabetes Federation Diabetes Atlas, 9th edition. Diabetes Res Clin Pract. 2019;157:107843. 10.1016/j.diabres.2019.107843 . 10.1016/j.diabres.2019.107843 31518657

[2] Wang C, Li J, Xue H, Li Y, Huang J, Mai J (2015). Type 2 diabetes mellitus incidence in Chinese: contributions of overweight and obesity. Diabetes Res Clin Pract.

[3] Yang W, Lu J, Weng J, Jia W, Ji L, Xiao J (2010). Prevalence of diabetes among men and women in China. N Engl J Med.

[4] Lu B, Yang Y, Yang Z, Feng X, Wang X, Zhang Z, Hu R (2010). Insulin resistance in Chinese patients with type 2 diabetes is associated with C-reactive protein independent of abdominal obesity. Cardiovasc Diabetol.

[5] Lean MEJ, Leslie WS, Barnes AC, Brosnahan N, Thom G, McCombie L (2019). Durability of a primary care-led weight-management intervention for remission of type 2 diabetes: 2-year results of the DiRECT open-label, cluster-randomised trial. Lancet Diabetes Endocrinol.

[6] Bennett DA, Du H, Bragg F, Guo Y, Wright N, Yang L (2019). Physical activity, sedentary leisure-time and risk of incident type 2 diabetes: a prospective study of 512 000 Chinese adults. BMJ Open Diabetes Res Care.

[7] Mohanty SA, Woolhandler S, Himmelstein DU, Bor DH (2005). Diabetes and cardiovascular disease among Asian Indians in the United States. J Gen Intern Med.

[8] Yang J, Wang F, Wang J, Han X, Hu H, Yu C (2018). Using different anthropometric indices to assess prediction ability of type 2 diabetes in elderly population: a 5 year prospective study. BMC Geriatr.

[9] Lim RBT, Chen C, Naidoo N, Gay G, Tang WE, Seah D (2015). Anthropometrics indices of obesity, and all-cause and cardiovascular disease-related mortality, in an Asian cohort with type 2 diabetes mellitus. Diabetes Metab.

[10] Hlatky MA, Chung SC, Escobedo J, Hillegass WB, Melsop K, Rogers W (2010). The effect of obesity on quality of life in patients with diabetes and coronary artery disease. Am Heart J.

[11] Motamed N, Rabiee B, Keyvani H, Hemasi GR, Khonsari M, Saeedian FS (2016). The best obesity indices to discriminate type 2 diabetes mellitus. Metab Syndr Relat Disord.

[12] Zerga AA, Bezabih AM, Adhanu AK, Tadesse SE (2020). Obesity indices for identifying metabolic syndrome among type two diabetes patients attending their follow-up in Dessie referral hospital, north East Ethiopia. Diabetes Metab Syndr Obes.

[13] Wang S, Ma W, Yuan Z, Wang SM, Yi X, Jia H, Xue F (2016). Association between obesity indices and type 2 diabetes mellitus among middle-aged and elderly people in Jinan, China: a cross-sectional study. BMJ Open.

[14] Xiao X, Liu Y, Sun C, Gang X, Cheng J, Tian S (2015). Evaluation of different obesity indices as predictors of type 2 diabetes mellitus in a Chinese population. J Diabetes.

[15] Rezende FA, Ribeiro AQ, Mingoti SA, Pereira PF, Marins JC, Priore SE, Franceschini SC (2018). Anthropometric patterns of adiposity, hypertension and diabetes mellitus in older adults of Viçosa, Brazil: a population-based study. Geriatr Gerontol Int.

[16] de Oliveira CM, Pavani J, Krieger JE, de Oliveira AR, Mourão-Junior CA, da Costa Pereira A (2019). Body adiposity index in assessing the risk of type 2 diabetes mellitus development: the Baependi heart study. Diabetol Metab Syndr.

[17] Wei J, Liu X, Xue H, Wang Y, Shi Z (2019). Comparisons of visceral adiposity index, body shape index, body mass index and waist circumference and their associations with diabetes mellitus in adults. Nutrients..

[18] Bragg F, Tang K, Guo Y, Iona A, Du H, Holmes MV (2018). Associations of general and central adiposity with incident diabetes in Chinese men and women. Diabetes Care.

[19] Wang X, Xinge J (2020). Sample size estimation in clinical research: from randomized controlled trials to observational studies. Chest..

[20] Ma RCW (2018). Epidemiology of diabetes and diabetic complications in China. Diabetologia..

[21] Chinese Guidelines for the Prevention and treatment of Type 2 diabetes (2017 edition). Chinese Journal of Diabetes. 2015;10(01):4–67.

[22] Chen CM, Kong LZ. Chinese guidelines for the prevention and control of overweight and obesity in adults. Beijing: People's medical Publishing House, 2006:3.

[23] Yu K, Li S, Zheng LB, Li Y, Bao J, Zhang X (2019). Research on the applicability of BAI to obesity evaluation of Chinese rural Han adults. J Tianjin Normal University.

[24] Wickramasinghe VP, Arambepola C, Bandara P, Abeysekera M, Kuruppu S, Dilshan P, Dissanayake BS (2017). Insulin resistance in a cohort of 5-15 year old children in urban Sri Lanka. BMC Res Notes.

[25] Dong B, Wang Z, Arnold LW, Song Y, Wang HJ, Ma J (2016). Simplifying the screening of abdominal adiposity in Chinese children with waist-to-height ratio. Am J Hum Biol.

[26] Tarleton HP, Smith LV, Zhang ZF, Kuo T (2014). Utility of anthropometric measures in a multiethnic population: their association with prevalent diabetes, hypertension and other chronic disease comorbidities. J Community Health.

[27] He Y, Zhai F, Ma G, Feskens EJ, Zhang J, Fu P, Van't Veer P, Yang X (2009). Abdominal obesity and the prevalence of diabetes and intermediate hyperglycaemia in Chinese adults. Public Health Nutr.

[28] Yuan Y, Xie H, Sun L, Wang B, Zhang L, Han H, Yao R, Sun Y, Fu L (2020). A novel Indicator of Children's lipid accumulation product associated with impaired fasting glucose in Chinese children and adolescents. Diabetes Metab Syndr Obes.

[29] Nyamdorj R, Qiao Q, Söderberg S, Pitkäniemi JM, Zimmet PZ, Shaw JE (2009). BMI compared with central obesity indicators as a predictor of diabetes incidence in Mauritius. Obesity (Silver Spring).

[30] Karter AJ, D'Agostino RB, Mayer-Davis EJ, Wagenknecht LE, Hanley AJ, Hamman RF (2005). Abdominal obesity predicts declining insulin sensitivity in non-obese normoglycaemics: the insulin resistance atherosclerosis study (IRAS). Diabetes Obes Metab.

[31] Gray RS, Fabsitz RR, Cowan LD, Lee ET, Howard BV, Savage PJ (1998). Risk factor clustering in the insulin resistance syndrome. The strong heart study. Am J Epidemiol.

[32] Caspard H, Jabbour S, Hammar N, Fenici P, Sheehan JJ, Kosiborod M (2018). Recent trends in the prevalence of type 2 diabetes and the association with abdominal obesity lead to growing health disparities in the USA: an analysis of the NHANES surveys from 1999 to 2014. Diabetes Obes Metab.

[33] Kita S, Maeda N, Shimomura I (2019). Interorgan communication by exosomes, adipose tissue, and adiponectin in metabolic syndrome. J Clin Invest.

[34] Henneman P, Janssens AC, Zillikens MC, Frolich M, Frants RR, Oostra BA (2010). Menopause impacts the relation of plasma adiponectin levels with the metabolic syndrome. J Intern Med.

[35] Kahn Steven E, Hull Rebecca L, Utzschneider KM (2006). Mechanisms linking obesity to insulin resistance and type 2 diabetes. Nature..

[36] Alokail MS, Al-Daghri NM, Al-Attas OS, Hussain T (2009). Combined effects of obesity and type 2 diabetes contribute to increased breast cancer risk in premenopausal women. Cardiovasc Diabetol.

[37] Won KB, Hur SH, Cho YK, Yoon HJ, Nam CW, Kim KB (2015). Comparison of 2-year mortality according to obesity in stabilized patients with type 2 diabetes mellitus after acute myocardial infarction: results from the DIAMOND prospective cohort registry. Cardiovasc Diabetol.

[38] Tchernof A, Després JP (2013). Pathophysiology of human visceral obesity: an update. Physiol Rev.

[39] Wan H, Wang Y, Xiang Q, Fang S, Chen Y, Chen C (2020). Associations between abdominal obesity indices and diabetic complications: Chinese visceral adiposity index and neck circumference. Cardiovasc Diabetol.

[40] Suarez EC (2008). Self-reported symptoms of sleep disturbance and inflammation, coagulation, insulin resistance and psychosocial distress: evidence for gender disparity. Brain Behav Immun.

[41] Zh Zhang JG, Wang ZH, Wang HJ, Du WW, Su C, Zhang J (2015). Dietary patterns and their associations with general obesity and abdominal obesity among young Chinese women. Eur J Clin Nutr.

[42] Bergman RN. A better index of body adiposity. Obesity (Silver Spring). 2012;20(6):1135. 10.1038/oby.2011.38 . 10.1038/oby.2012.99 22627975

[43] Schulze MB, Thorand B, Fritsche A, Häring HU, Schick F, Zierer A (2012). Body adiposity index, body fat content and incidence of type 2 diabetes. Diabetologia..

[44] Freedman DS, Thornton JC, Pi-Sunyer FX, Heymsfield SB, Wang J, Pierson RN Jr, Blanck HM, Gallagher D. The body adiposity index (hip circumference ÷ height(1.5)) is not a more accurate measure of adiposity than is BMI, waist circumference, or hip circumference. Obesity (Silver Spring). 2012;20(12):2438–44. 10.1038/oby.2012.81 . 10.1038/oby.2012.81 PMC3477292 22484365

